# Effective Preparation of FFPE Tissue Samples for Preserving Appropriate Nucleic Acid Quality for Genomic Analysis in Thyroid Carcinoma

**DOI:** 10.1007/s12022-024-09838-9

**Published:** 2024-11-19

**Authors:** Yoichiro Okubo, Nagisa Toyama, Rika Kasajima, Soji Toda, Hiroyuki Hayashi, Emi Yoshioka, Kota Washimi, Shinya Sato, Yukihiko Hiroshima, Chie Hasegawa, Shu Yuguchi, Mei Kadoya, Hiroto Narimatsu, Katsuhiko Masudo, Hiroyuki Iwasaki, Tomoyuki Yokose, Yohei Miyagi

**Affiliations:** 1 Department of Pathology, Kanagawa Cancer Center, 2-3-2, Nakao, Asahi-Ku, Yokohama, Kanagawa 241-8515 Japan; 2 Molecular Pathology and Genetics Division, Kanagawa Cancer Center Research Institute, 2-3-2, Nakao, Asahi-Ku, Yokohama, Kanagawa 241-8515 Japan; 3 Department of Endocrine Surgery, Kanagawa Cancer Center, 2-3-2, Nakao, Asahi-Ku, Yokohama, Kanagawa 241-8515 Japan; 4 Department of Pathology, Yokohama Municipal Citizen’s Hospital, 1-1 Mitsuzawanishimachi, Kanagawa-Ku, Yokohama, Kanagawa 221-0855 Japan; 5 Department of Cancer Genome Medicine, Kanagawa Cancer Center, 2-3-2, Nakao, Asahi-Ku, Yokohama, Kanagawa 241-8515 Japan; 6 Advanced Cancer Therapeutics Division, Kanagawa Cancer Center Research Institute, 2-3-2, Nakao, Asahi-Ku, Yokohama, Kanagawa 241-8515 Japan; 7 Department of Genetic Medicine, Cancer Prevention and Cancer Control Division, Kanagawa Cancer Center Research Institute, 2-3-2, Nakao, Asahi-Ku, Yokohama, Kanagawa 241-8515 Japan; 8 Cancer Prevention and Cancer Control Division, Kanagawa Cancer Center Research Institute, 2-3-2, Nakao, Asahi-Ku, Yokohama, Kanagawa 241-8515 Japan

**Keywords:** Thyroid carcinoma, Formalin-fixed paraffin-embedded, Nucleic acid quality, Genomic analysis

## Abstract

Formalin-fixed paraffin-embedded (FFPE) tissue samples are important for genomic analysis of thyroid carcinomas, particularly for various molecularly targeted therapies. Therefore, this study developed and validated a technique for preparing FFPE tissue samples that preserves nucleic acid quality, which is fundamental for precise genomic analysis, more effectively than conventional methods. We analyzed surgically resected thyroid gland tumors, lymph node metastases, and separately fixed tumor samples to optimize formalin fixation and assess the influence of specimen type and preparation methods on nucleic acid quality. We assessed several quality indicators, including the DNA integrity number, cycle threshold ratio, RNA integrity number, and DV200. Separately fixed tumor samples consistently exhibited higher DNA and RNA quality than conventionally processed samples. Additionally, lymph node metastases often exhibit nucleic acid quality matching or exceeding that of thyroid gland tumors, highlighting their potential reliability for genomic analysis. These findings suggest the utility of various specimen types for the comprehensive genetic profiling of thyroid carcinomas. In conclusion, this study demonstrated that preparing separately fixed tumor samples is an effective method for preserving DNA and RNA quality for genomic analyses. Biopsy punches enable specimen collection at various facilities, including those without the ability to handle frozen specimens. This contributes to the development of a method for obtaining high-quality pathological samples that can be widely used in general medical practice. Moreover, lymph node metastases often exhibit nucleic acid quality equal to or superior to that of thyroid gland tumors, highlighting their potential as acceptable sources for genomic analyses.

**Supplementary Information:**

The online version contains supplementary material available at 10.1007/s12022-024-09838-9.

## Introduction

Recently, significant advances have been made in the field of genomic cancer medicine. This has facilitated the development of molecular-targeted therapies for various tumors, including thyroid carcinoma [1–3]. These therapies require accurate companion diagnostic tests to detect mutations or fusion genes in targeted patients [4]. Formalin-fixed paraffin-embedded (FFPE) tissue samples are commonly used for genomic analysis [5, 6]. Although general pathological diagnosis typically relies on the primary lesion, whether the primary lesion or metastases from the same tumor are more suitable for genomic analysis to assess the efficacy of molecularly targeted therapies remains an unanswered question. Although the Japanese Society of Pathology has issued broad guidelines for all organs [7], such as fixing surgical specimens in formalin within 1 h (and no later than 3 h) after removal, keeping the ischemic time as short as possible, and maintaining fixation time between 6 and 48 h, processing thyroid cancer specimens still lacks standardized methodology. A study using both thyroid and lymph node metastases as sources for next-generation sequencing (NGS) analysis [8] found no significant differences in nucleic acid quality between the two sources. However, as such studies are limited, further verification is required to determine the most appropriate source for these analyses. In this study, we aimed to evaluate nucleic acid quality in processed specimens obtained from thyroid carcinoma cases and propose criteria for selecting the most appropriate specimens for genomic analysis based on these findings.

## Materials and Methods

### Included Cases

This study included surgical excision specimens obtained from patients with thyroid carcinoma at Kanagawa Cancer Center between May 2023 and February 2024. Specimens from patients diagnosed preoperatively with benign lesions and those without any specimen processing by a pathologist were excluded from the study.

### Specimen Processing and Sectioning

**Fig. 1:**
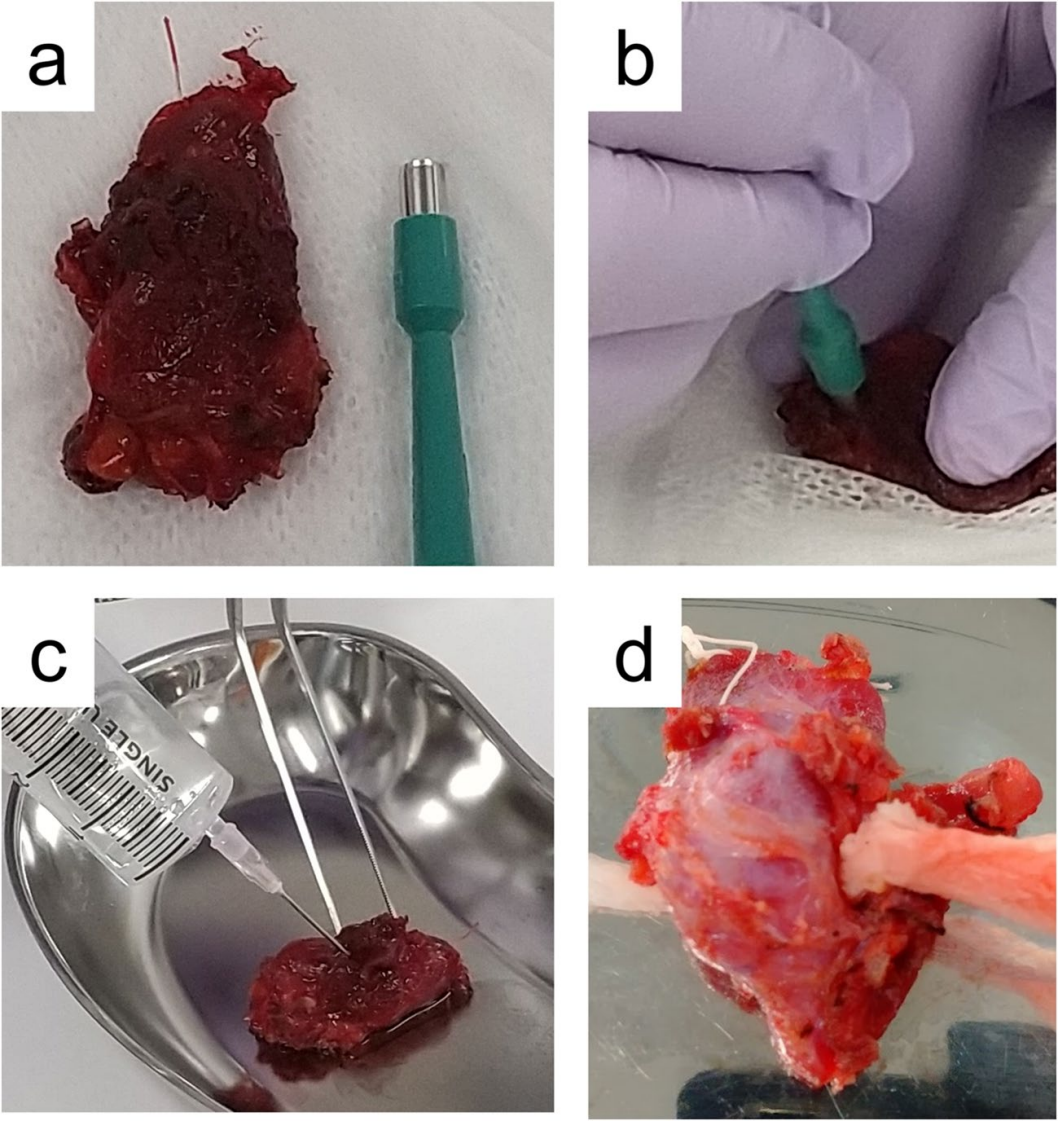
Processing and fixation techniques for thyroid gland specimens. **a**, **b** Upon arrival at the Department of Pathology, the thyroid gland was reviewed by a pathologist. Selected areas of the thyroid gland were excised by using a biopsy punch. Separately fixed tumor samples were immediately placed in formalin to preserve their molecular integrity. **c** Formalin was directly injected into the thyroid gland to ensure the deep and uniform penetration of the fixative throughout the sample. **d** To improve formalin fixation, gauze was threaded through the biopsy punch sites in the gland. This method enhances formalin permeation from the interior outward, thereby optimizing fixation

After surgical excision, the specimens were immediately delivered to the Department of Pathology and verified against the order form by clinical laboratory technicians. In this study, we introduced the concept of “separately fixed tumor samples.” These are small portions of the tumor, 3–5 mm in diameter, that are immediately fixed in 10% neutral buffered formalin upon receipt of the specimen, separate from the main tumor mass. This approach aimed to optimize nucleic acid preservation by ensuring rapid and uniform fixation. Two pathologists (YO and EY) reviewed each specimen and obtained a “separately fixed tumor sample” for the lesion using a biopsy punch needle (KAI Biopsy Punch, KAI Corporation, Tokyo, Japan) with a diameter of 3–5 mm, which was immediately fixed in 10% neutral buffered formalin. However, if the lesion was < 1 cm in diameter upon gross inspection by pathologists, a separately fixed tumor sample was not obtained to preserve the tissue for routine diagnosis. This assessment was based on pathologists’ subjective visual evaluation at the time of specimen receipt. Specimens from patients who consented to have tissue samples frozen had a portion of the separately fixed tumor sample reserved for freezing. The remaining thyroid gland was further processed by inserting a gauze into the biopsy punch site, injecting formalin, and submerging it in formalin using a vacuum fixation device to enhance fixation (THW-100, Azumaya, Tokyo, Japan) [9, 10]. This procedure is summarized in Fig. 1. Similarly, all lymph nodes excised during surgery were immediately placed in formalin containers in the operating room and then delivered to the pathology department. For lymph nodes with clearly visible large metastases, incisions (referred to as incisions made with scalpel cutting, distinct from the biopsy punch) were made to enhance formalin penetration, and separately fixed tumor samples were obtained, similar to those obtained from thyroid gland tumors. Formalin-fixed specimens were sectioned the following day. The specimens were then checked for fixation status and additional formalin fixation was performed as required. “Separately fixed tumor samples” were removed from formalin at 9:00 a.m. the following day to ensure a complete fixation process based on our experience that overnight formalin fixation is sufficient for separately fixed tumor samples (based on our laboratory practice). This standardized overnight fixation period allows for consistent and optimal formalin penetration, which is crucial for preserving the nucleic acid quality.

### Selecting Slides for Nucleic Acid Quality Analysis

Pathologists assessed H&E-stained slides of the thyroid gland, lymph nodes, and separately fixed tumor samples to identify the tumor content suitable for nucleic acid quality analysis. The selection criteria adhered to the Oncomine Dx Target Test (ODxTT; Thermo Fisher Scientific, Waltham, MA, USA), an NGS-based method approved for thyroid carcinoma in Japan. The system generally requires a minimum tumor content of 20% and 15 unstained slides of 4–5 µm thickness each. Although macrodissection is acceptable for clearing the minimum tumor content set, tissue areas of more than 4 mm^2^ should be prepared (the number of slides may be adjusted based on the tissue sample area). Tissue samples that underwent ethylenediaminetetraacetic acid- or hydrochloric acid-based decalcification were excluded from the study. Once these criteria were satisfied, the thyroid gland tumor sections closest to the biopsy punch site with optimal formalin fixation were selected for analysis. The specimens with the largest metastatic size were selected for lymph node metastasis. Clinicopathological data, including age, sex, tumor diameter, size of lymph node metastasis, TNM classification, and formalin fixation time, were recorded and prepared for nucleic acid quality analysis to ensure that it could be initiated within 2 months of surgical intervention.

### DNA Extraction and Quality Assessment

DNA was extracted from unstained FFPE sections using a Qiagen QIAamp DNA FFPE advanced UNG kit (Qiagen, Tegelen, Netherland). DNA concentrations were determined using a NanoDrop microvolume spectrophotometer (Thermo Fisher Scientific) and the Qubit fluorometric method with a kit (Thermo Fisher Scientific). DNA samples with A260/A280 ratios ranging from 1.8 to 2.0, and with concentrations greater than 20 ng/µL were further subjected to DNA quality assessment. To assess the integrity of the DNA samples, the DNA integrity number (DIN) was measured using an Agilent 4200 TapeStation system (Agilent Technologies, Santa Clara, CA, USA) and a Genomic DNA Screen Tape assay (Agilent) according to the manufacturer’s protocol. In addition, the short-to-long cycle threshold (S/L Ct) ratio was determined by performing a TaqMan® PCR assay with the LightCycler® 480 real-time PCR device (Hoffmann-La Roshe Ltd, Basel, Switzerland). Briefly, two DNA fragments with 87 base pairs (short) synthesized using the TaqMan® RNase P Detection Kit (Thermo Fisher Scientific) and 256 base pairs (long) synthesized with the TaqMan® MGB gene expression assay kit (Thermo Fisher Scientific) were amplified using the LightCycler® TaqMan Master (Hoffmann-La Roshe Ltd), and Ct values for both the sample specimens and the human control DNA were recorded. The S/L Ct ratio was calculated by dividing the Ct value of the short assay by that of the long assay. This ratio for each sample was then normalized against the data of the control to obtain relative DNA quality, with values close to or greater than 1 indicating a quality comparable to or exceeding that of the control. The TaqMan® PCR assay was performed using 10 ng of DNA. The PCR conditions used are listed in Table 1.

**Table 1:** PCR conditions for DNA quality assessment

*Step*	*Temperature, °C*	*Time*	*Number of cycles*
*Enzyme activation and thermosetting*	95	3 min	1
*Denaturation*	95	10 s	70 (Until the plateau is reached)
*Annealing/extension*	60	15 s	
*Extension*	72	30 s	
*Hold*	4		1

This table outlines the thermocycling parameters used during PCR amplification to assess DNA quality in FFPE tissue sections. The steps include initial enzyme activation and thermosetting, followed by cycles of denaturation, annealing, extension, and a final hold at a low temperature to stabilize the amplified DNA products

### RNA Extraction and Quality Assessment

RNA was extracted from unstained FFPE sections using the Qiagen RNeasy FFPE kit (Qiagen), according to the manufacturer’s protocol. RNA concentrations were assessed spectrophotometrically using NanoDrop and the Qubit RNA BR assay kit (Thermo Fisher Scientific). RNA samples with A260/A280 ratios ranging from 1.8 to 2.0, and with concentrations greater than 20 ng/µL were further subjected to RNA quality assessment, and the RNA integrity number (RIN) and DV200 values were measured with Agilent 4200 TapeStation system using High Sensitivity RNA Screen Tape assay (Agilent). Due to the lack of established qPCR protocols for analyzing RNA quality in our hospital, this study used electrophoretic assessments (RIN and DV200) to evaluate RNA quality.

### Statistical Analysis

Univariate analyses were performed to assess the distribution of the continuous variables. The Shapiro–Wilk test was initially conducted to determine whether the data were parametric or non-parametric. Based on these results, statistical comparisons were made using the *t*-test for parametric data and the Mann–Whitney U test for non-parametric data. The correlation analyses were performed using Pearson’s correlation coefficient for parametric data, and Spearman’s rank correlation coefficient for non-parametric data. Additionally, the Kruskal–Wallis test, followed by Bonferroni correction, was performed to compare nucleic acid quality across the three specimen groups: thyroid gland lesions, lymph node metastases, and separately fixed tumor samples. Univariate and multiple regression analyses were performed to evaluate the relationship between the histological features and nucleic acid quality parameters for each specimen type. All available samples were included in the statistical analyses, without predetermined sample size calculations. All statistical analyses were conducted using IBM SPSS Statistics (IBM Corp., Armonk, NY, USA). Statistical significance was set at *P* < 0.05.

### Ethics Consideration

This study was conducted in accordance with ethical standards and approved by the Kanagawa Cancer Center Ethics Committee (Approval No.: 2022-Eki-121).

## Results

### Clinicopathological Information

**Fig. 2:**
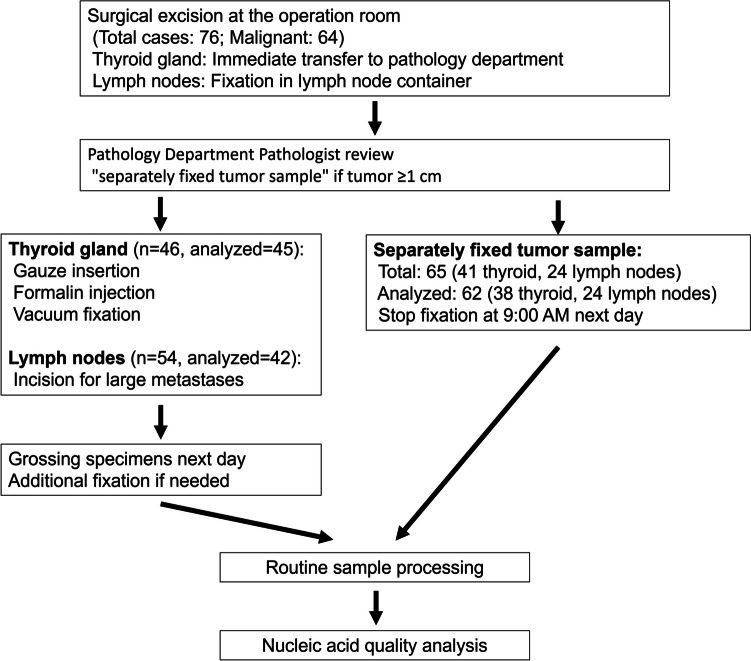
Flowchart of specimen processing for nucleic acid quality analysis in thyroid carcinoma cases. This flowchart shows the processing of surgical specimens from patients with thyroid carcinoma. It details the handling of thyroid gland tumors and lymph node metastases, including the preparation of “separately fixed tumor samples.” The chart outlines the workflow from surgical excision to nucleic acid quality analysis and shows the number of specimens processed at each stage. For thyroid gland tumors, 45 of the 46 cases were included in the analysis. For lymph nodes, although specimens were collected from all 54 cases, 42 were included in the final analysis. Of the 65 separately fixed tumor samples collected, 62 were suitable for analysis after meeting the study criteria, including sufficient tumor content. In this study, cases with preoperatively diagnosed benign lesions were excluded

During the study period, 76 surgical interventions were performed at the Department of Endocrine Surgery of Kanagawa Cancer Center. A total of 64 cases were diagnosed as malignant tumors: 59 papillary thyroid carcinomas (PTCs), 3 follicular thyroid carcinomas (FTCs), 1 poorly differentiated thyroid carcinoma (PDTC), and 1 anaplastic thyroid carcinoma (ATC). The remaining 12 patients had benign disease, including adenomatous goiter (*n* = 8), Basedow’s disease (*n* = 3), and Plummer’s disease (*n* = 1). Separately fixed tumor samples that met the criteria were successfully obtained from either thyroid gland tumors or lymph node metastases in 54 of the 64 malignant cases. Specifically, separately fixed tumor samples were obtained from 41 thyroid gland tumors, of which 38 met the inclusion criteria. The three excluded samples had insufficient numbers of tumor cells, often due to fibrosis and/or calcification. Twenty-four separately fixed tumor samples were obtained from the largest metastatic foci of lymph node metastases in 22 patients, resulting in 62 separately fixed tumor samples from 54 patients. Surgical techniques involved 37 initial thyroidectomies and 9 completion thyroidectomies with lymph node dissection for a total of 46 cases. In addition, lymph nodes alone were resected in 8 other cases, resulting in a total of 54 cases in which the lymph nodes were examined. In this study, 45 cases of thyroid tumors and 42 cases of lymph node metastases met the criteria for the analysis. Macrodissection was performed in 18/45 cases with thyroid gland tumors, 3/42 cases with lymph node metastases, and 8/62 cases with separately fixed tumor samples to meet the minimum 20% tumor content requirement for nucleic acid quality analysis. The basic clinicopathological findings are presented in Table 2. The specimen processing workflow is illustrated in Fig. 2. Detailed clinicopathological information for all 54 cases is provided in Supplementary Table 1.

**Table 2:** Clinicopathological findings of 54 patients with thyroid carcinoma

Category	Description
Age (years, median, range)	60 (24–81)
Male-to-female ratio	20:34
Histological type	PTC: 50, FTC: 2, PDTC: 1, ATC: 1
pT stage	pT1: 14, pT2: 7, pT3: 13, pT4: 3
pN stage	pN0: 7, pN1a: 23, pN1b: 20, distant lymph nodes: 5
cM stage	cM0: 41, cM1: 13 (lung: 7, distant lymph nodes: 5, bone: 2, skin: 1)
Thyroid gland tumor size (mm, median, range)	21 (5–62)
LN metastasis size (mm, median, range)	10 (0.07–75)
Formalin fixation time of thyroid gland and lymph nodes (hours, median, range)	41.5 (20.3–93.4)
Formalin fixation time of separately fixed tumor samples (hours, median, range)	21.3 (15.7–23.9)

In this study, 54 cases of thyroid carcinoma were evaluated. Thyroid gland tumor size refers to the diameter of the tumor or the size of recurrent metastases in the thyroid gland. LN metastasis size indicates the size of the largest metastasis. Of the 54 cases, only lymph node metastases were resected in 8 cases, resulting in 46 thyroidectomies. However, 9 patients underwent complete complementary thyroidectomies, allowing pT staging in 37 cases. Some specimens were from lymph nodes only but were classified as pN1a, pN1b, or treated as distant metastases. The formalin fixation time was standardized for the thyroid and lymph nodes

*PTC* papillary thyroid carcinoma, *FTC* follicular thyroid carcinoma, *PDTC* poorly differentiated thyroid carcinoma, *ATC* anaplastic thyroid carcinoma, *LN* lymph node

### Formalin Fixation Time and Tumor Diameter

The median formalin fixation time was 41.6 h (range, 20.3–93.4 h) for thyroid gland tumors and lymph nodes and 21.3 h (range, 15.7–23.9 h) for separately fixed tumor samples. The median diameter of the thyroid gland tumors was 21 mm (range, 5–62 mm), and that of the lymph node metastases was 10 mm (range, 0.07–75 mm). These findings are summarized in Table 2.

### Nucleic Acid Quality Assessment

Overall, a significant positive correlation was observed between DIN and S/L Ct ratio (Spearman’s correlation *ρ* = 0.707, *P* < 0.001) and between RIN and DV200 (Spearman’s correlation *ρ* = 0.361, *P* < 0.001).

Formalin fixation time was significantly negatively correlated with both DIN and S/L Ct ratio of thyroid gland tumors (Spearman’s correlation *ρ* = − 0.342, *P* = 0.023; *ρ* = − 0.445, *P* = 0.002). Conversely, formalin fixation time was significantly positively correlated with the RIN of thyroid gland tumors (*ρ* = 0.415, *P* = 0.005). No significant correlations were found for DV200 across thyroid gland tumors and lymph node metastases. These findings are summarized in Table 3.

**Table 3:** Results of nucleic acid quality evaluation of thyroid gland tumors, lymph node metastases, and separately fixed tumor samples

Category	Thyroid gland lesion	Lymph node metastasis	Separately fixed tumor sample
Number of samples	45	42	62
DIN, median (range)	3.2 (1.8–5.5)	3.8 (2.1–5.7)	4.7 (2.9–6)
S/L Ct ratio, median (range)	0.911 (0.854–0.959)	0.928 (0.815–0.96)	0.942 (0.867–0.984)
RIN, median (range)	2.5 (1–5.2)	2.4 (1.4–4)	2.1 (1.4–3.2)
DV200, median (range)	60.1 (23.9–87)	60.8 (19.2–87.5)	72.2 (26.8–87.3)

This table shows the nucleic acid quality assessment results for the 54 thyroid carcinoma cases divided into three categories: thyroid tumors, lymph node metastases, and separately fixed tumor samples. It shows the number of samples analyzed and the median values with corresponding ranges for four nucleic acid quality categories: DNA integrity number, short-to-long cycle threshold ratio, RNA integrity number, and DV200 values, indicating nucleic acid quality across sample types

*DIN* DNA integrity number, *S/L Ct ratio* short-to-long Ct ratio, *RIN* RNA integrity number

**Fig. 3:**
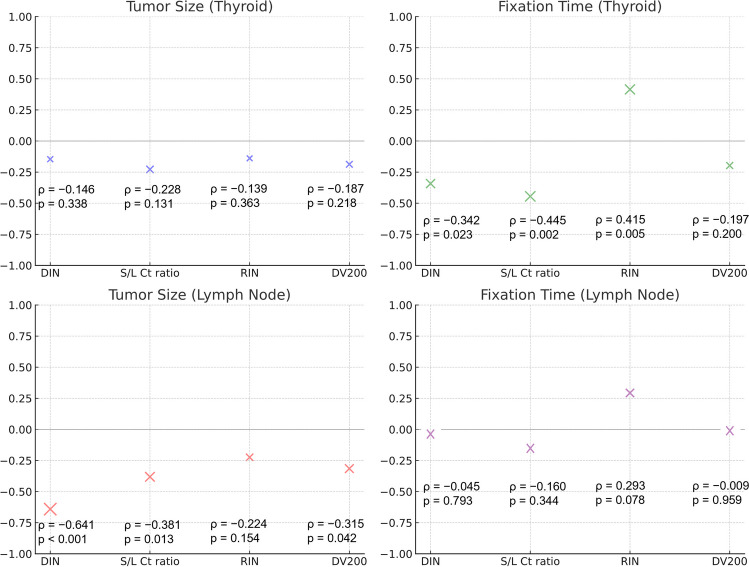
Correlation of nucleic acid quality with size and fixation time in thyroid tumors and lymph node metastases. This figure summarizes the Spearman’s correlation coefficients (*ρ*) and their significance (*P*-values) between tumor size or formalin fixation time and nucleic acid quality parameters (DIN, S/L Ct ratio, RIN, and DV200) for thyroid gland tumors and lymph node metastases. In thyroid gland tumors, tumor size was not significantly correlated with nucleic acid quality, whereas formalin fixation time was negatively correlated with both DIN and S/L Ct ratio. In lymph node metastases, metastasis size was negatively correlated with DIN, S/L Ct ratio, and DV200, whereas formalin fixation time was not significantly correlated with these nucleic acid quality parameters. Abbreviations: DIN, DNA integrity number; S/L Ct ratio, short-to-long Ct ratio; RIN, RNA integrity number

No significant correlation was detected between tumor size and nucleic acid quality parameters (DIN, S/L Ct ratio, RIN, and DV200) in thyroid gland tumors. For lymph node metastases, the size of metastases showed strong negative correlations with DIN, S/L Ct ratio, and DV200 (*ρ* = − 0.641, *P* < 0.001; *ρ* = − 0.381, *P* = 0.013; *ρ* = − 0.315, *P* = 0.042), respectively. These findings are summarized in Fig. 3.

Comparative analysis by specimen origin for separately fixed tumor samples showed no significant differences in DIN, RIN, S/L Ct ratio, or DV200 between the 38 samples collected from thyroid gland tumors and 24 from lymph node metastases (all assessed using the Mann–Whitney *U* test, *P* > 0.05).

### Summary of Nucleic Acid Quality Across Different Specimens

**Fig. 4:**
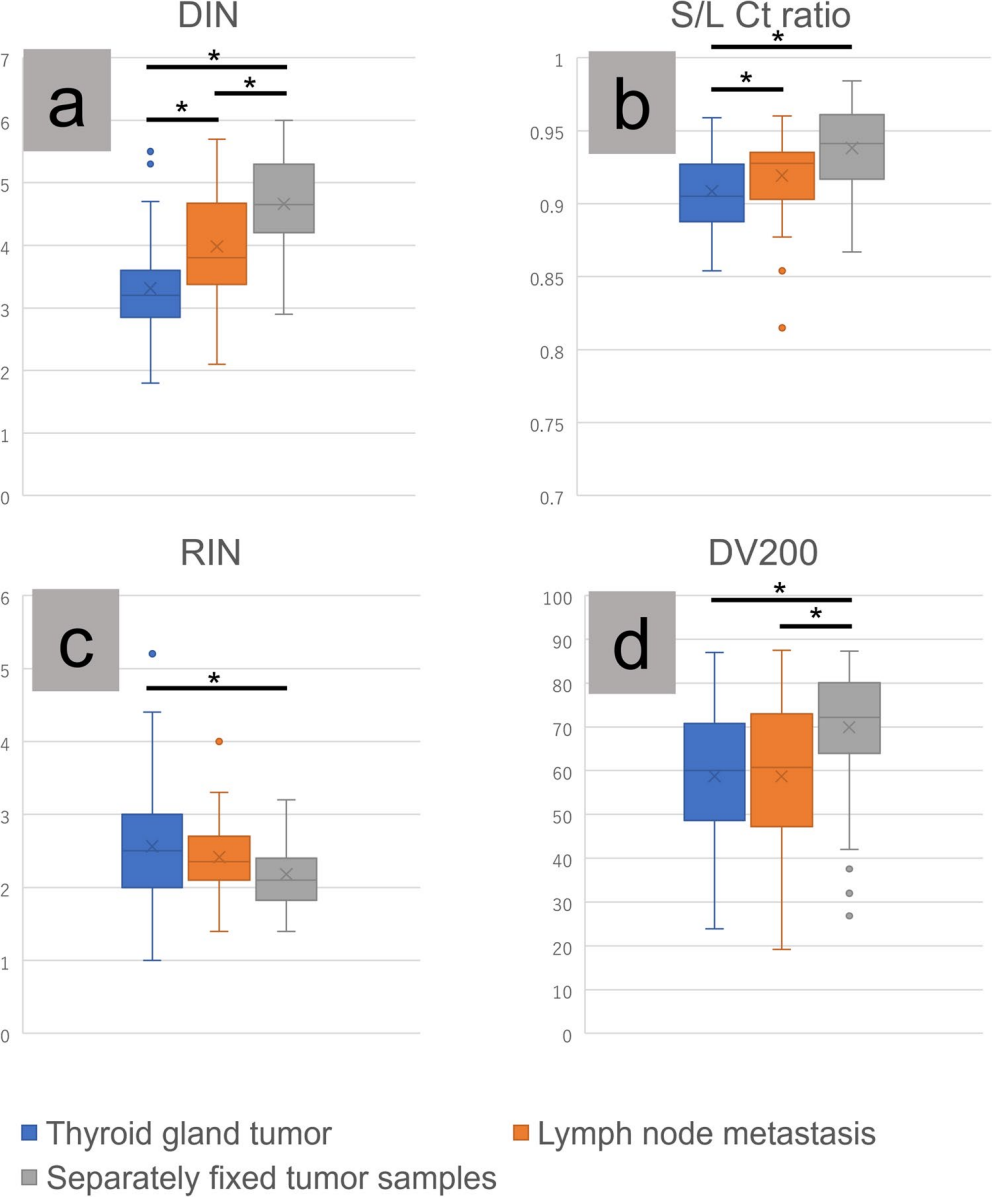
Comparative analysis of nucleic acid quality in thyroid specimens. This box-and-whisker diagram illustrates the nucleic acid quality parameters in thyroid gland tumors, lymph node metastases, and separately fixed tumor samples. **a** Separately fixed tumor samples demonstrated a significantly higher DNA integrity number (DIN) than both thyroid gland tumors and lymph node metastases, with lymph node metastases also exhibiting a higher DIN than thyroid gland tumors. **b** Both separately fixed tumor samples and lymph node metastases exhibited higher short-to-long cycle threshold ratios than thyroid gland tumors, although no significant differences were noted between separately fixed tumor samples and lymph node metastases. **c** RNA integrity number was lower in separately fixed tumor samples than in thyroid gland tumors. **d** DV200 values were significantly higher in separately fixed tumor samples than in thyroid gland tumors and lymph node metastases, with no significant differences observed between thyroid gland tumors and lymph node metastases

- DIN: Separately fixed tumor samples showed the highest median DIN values, significantly exceeding those of thyroid gland tumors and lymph node metastases.
- S/L Ct ratio: Separately fixed tumor samples also showed the highest median S/L Ct ratio, exceeding those of thyroid gland tumors and lymph node metastases.
- RIN: Separately fixed tumor samples had lower median values of RIN than thyroid gland tumors and were also lower than those for lymph node metastases, although the latter difference was not significant.
- DV200: Separately fixed tumor samples showed the highest median DV200 values, exceeding those of both thyroid gland tumors and lymph node metastases; no significant difference was observed between the latter two. The data analyzed for the four nucleic acid parameters (DIN, S/L Ct ratio, RIN, and DV200) are summarized in Fig. 4 and Tables 3 and 4.

**Table 4:** Nucleic acid quality parameters across the specimens

	DNA integrity number	Short-to-long Ct ratio	RNA integrity number	DV200
Thyroid gland lesion vs. lymph node metastasis	*P* = 0.003	*P* = 0.118	*P* = 1	*P* = 1
Lymph node metastasis vs. separately fixed tumor sample	*P* = 0.001	*P* = 0.008	*P* = 0.063	*P* = 0.004
Thyroid gland lesion vs. separately fixed tumor sample	*P* < 0.001	*P* < 0.001	*P* = 0.009	*P* = 0.001

This table shows *P*-values from statistical comparisons of nucleic acid quality parameters among thyroid gland tumors, lymph node metastases, and separately fixed tumor samples. Statistical significance was determined using the Kruskal–Wallis test, with Bonferroni correction applied for multiple comparisons. Statistical significance was set at* P* < *0.05*

### Histological Features and Nucleic Acid Quality

We analyzed the histological features and their impact on nucleic acid quality across specimen types. These features are summarized in Table 5. In thyroid gland tumors, multiple regression analysis showed that the presence of cystic changes was positively associated with the S/L Ct ratio (*β* = 0.373, *P* = 0.038), while the lymphocyte ratio was negatively correlated with DV200 (*β* = − 0.535, *P* = 0.013). For lymph node metastases, the fibrosis ratio negatively affected the S/L Ct ratio (*β* = − 0.705, *P* = 0.001). Macrodissection was associated with an improved RIN (*β* = 0.427, *P* = 0.010). In separately fixed tumor samples, the presence of cystic components significantly positively affected both DIN (*β* = 0.419, *P* = 0.001) and the S/L Ct ratio (*β* = 0.346, *P* = 0.011). No significant differences in nucleic acid quality were observed between *BRAF* immunohistochemistry-positive and immunohistochemistry-negative samples across all specimen types. The findings are summarized in Table 6 and Supplementary Table 2.

**Table 5:** Histological features of thyroid gland tumors, lymph node metastases, and separately fixed tumor samples

	Thyroid gland tumors (*n* = 45)	Lymph node metastases (*n* = 42)	Separately fixed tumor samples (*n* = 62)
Tumor content (%, median)	40	35	60
Fibrosis ratio (% area, median)	30	10	20
Lymphocyte ratio (%, median)	20	40	10
Cystic change (present: absent)	12:33	9:33	14:48
Macrodissection (performed: not performed)	18:27	3:39	8:54

This table summarizes the histological features of thyroid gland tumors, lymph node metastases, and separately fixed tumor samples. Tumor content represents the percentage of tumor cells. Fibrosis ratio is presented as the median percentage of fibrotic area. Lymphocyte ratio indicates the percentage of lymphocyte nuclei relative to total cell nuclei. The presence of cystic changes and the use of macrodissection are presented as ratios (present: absent or performed: not performed, respectively)

**Table 6:** Significant associations between histological features and nucleic acid quality parameters

Specimen type	Feature	Parameter	Analysis type	*P*-value	Coefficient
Thyroid gland tumors	Lymphocyte ratio	DV200	Multiple regression	0.013	*β* = − 0.535
	Cystic change	Short-to-long Ct ratio	Multiple regression	0.038	*β* = 0.373
	Lymphocyte ratio	RNA integrity number	Spearman correlation	0.038	*r* = − 0.310
	Lymphocyte ratio	DV200	Spearman correlation	0.032	*r* = − 0.320
	Cystic change	Short-to-long Ct ratio	Mann–Whitney *U* test	0.049	N/A
Lymph node metastases	Fibrosis ratio	Short-to-long Ct ratio	Multiple regression	0.001	*β* = − 0.705
	Macrodissection	RNA integrity number	Multiple regression	0.01	*β* = 0.427
	Tumor content	DNA integrity number	Spearman correlation	0.002	*r* = − 0.469
	Tumor content	Short-to-long Ct ratio	Spearman correlation	0.018	*r* = − 0.364
	Tumor content	DV200	Spearman correlation	0.003	*r* = − 0.451
	Fibrosis ratio	DNA integrity number	Spearman correlation	0.019	*r* = − 0.362
	Lymphocyte ratio	DNA integrity number	Spearman correlation	< 0.001	*r* = 0.534
	Lymphocyte ratio	Short-to-long Ct ratio	Spearman correlation	0.011	*r* = 0.388
	Lymphocyte ratio	DV200	Spearman correlation	0.01	*r* = 0.395
	Macrodissection	RNA integrity number	Mann–Whitney U test	0.041	N/A
	Macrodissection	DV200	Mann–Whitney U test	0.026	N/A
Separately fixed tumor samples	Cystic change	DNA integrity number	Multiple regression	0.001	*β* = 0.419
	Cystic change	Short-to-long Ct ratio	Multiple regression	0.011	*β* = 0.346
	Cystic change	DNA integrity number	Mann–Whitney U test	0.004	N/A
	Cystic change	Short-to-long Ct ratio	Mann–Whitney U test	0.015	N/A

This table presents only statistically significant associations (*P* < 0.05) between histological features and nucleic acid quality parameters. The results were primarily sorted by specimen type and then by analysis type (multiple regression followed by univariate analyses). β represents the standardized beta coefficient from the multiple regression analysis. For the Spearman correlation, r represents the correlation coefficient

## Discussion

With the increasing number of molecularly targeted therapies for thyroid carcinoma, the accurate identification of gene mutations and fusion genes is necessary to maximize therapeutic efficacy [4, 11, 12]. Particularly in thyroid carcinoma, some patients may exhibit late recurrence and/or distant metastasis [3, 13, 14]. Therefore, it is important to obtain long-term tissue samples that retain a high nucleic acid quality. In this study, we evaluated the nucleic acid quality of thyroid carcinoma specimens and investigated the utility of using separately fixed tumor samples. Given the typically high nucleic acid quality resulting from the effective formalin fixation of biopsy specimens [7], we prepared separately fixed tumor samples from the thyroid glands and lymph nodes immediately upon arrival at the Department of Pathology. Separately fixed tumor samples showed significantly higher values of DIN and S/L Ct ratio, the indicators of DNA quality, than conventionally processed thyroid tumors and lymph node metastases. These samples also showed significantly higher levels of DV200, an indicator of RNA quality. Considering their size of 3–5 mm and regulated fixation time in formalin, immediate and adequate fixation may have contributed to the preservation of nucleic acid quality by ensuring thorough and even formalin penetration without over- or under-fixation. Furthermore, the preparation of such samples can be seamlessly integrated into the routine workflow of pathological examinations, making it a highly valuable method for preserving nucleic acid quality in specimen samples, particularly in hospitals lacking frozen storage facilities. However, Hatanaka et al. reported higher DIN and DV200 values [8] than observed in the present study. This result may reflect differences in specimen characteristics and processing conditions at our specialized oncology hospital, which frequently handles advanced cases and large lymph node metastases.

Evaluation of the effect of formalin fixation time on nucleic acid quality in the present study showed that DNA quality (DIN and S/L Ct ratio) in thyroid gland tumors tended to decrease with longer fixation times. This finding aligns with those of the previous reports showing that excessive fixation negatively affects DNA quality [15, 16]. Such degradation can be attributed to the susceptibility of DNA to fragmentation during extended fixation periods, primarily due to cross-linking and chemical modifications that compromise the structural integrity of the DNA molecules [15]. These findings highlight the importance of optimizing fixation time to preserve DNA quality for subsequent genomic analyses. In contrast, no significant difference was observed between formalin fixation time and nucleic acid quality in lymph node metastasis. This lack of variation could be attributed to the anatomical and procedural differences between thyroid gland tumors and lymph nodes. For thyroid gland tumors, our fixation process involved a biopsy punch for lesions, followed by gauze insertion into the hollowed-out area, formalin injection, and subsequent submersion in formalin by vacuum-assisted fixation. While these methods aim to enhance formalin penetration, extended fixation times may result in over fixation, potentially affecting DNA quality. Conversely, lymph nodes did not undergo extensive processing in this study. Additionally, the presence of adipose tissue and capsules around the lymph nodes could impede the penetration of formalin solution [17]. These procedural and anatomical differences could explain why extended formalin fixation time alone did not lead to significant variation in nucleic acid quality in lymph node metastases.

In addition, we examined the quality of RNA in relation to specimen fixation time, which is known to promote the inactivation of RNases [18] and ensure more complete tissue fixation [19]. An appropriate fixation time can prevent RNA degradation and potentially improve RNA integrity preservation in properly fixed samples. In some cases, this may require longer fixation times than those optimal for DNA [19]. However, our findings reveal a more complex picture. The RIN values for the separately fixed tumor samples were lower than those for the other samples, in contrast to the parameters observed for DNA quality. Moreover, further analysis of histological features could not fully explain this discrepancy, suggesting that additional factors may be involved in determining the RNA quality in these samples. Although the RIN value is widely used as an indicator of RNA quality [20–22], it should be noted that separately fixed tumor samples exhibited the highest DV200 values, despite their lowest RIN values. Additionally, the RIN values of samples obtained from FFPE tissues were generally very low, indicating extended fragmentation. In contrast, high DV200 values, which represent the percentage of RNA fragments that are 200 bases or longer, suggest that a portion of RNA remains intact and available for NGS-based genomic analyses. Previous studies have reported that RIN values are generally lower in FFPE tissue samples derived from human surgical specimens [18]; however, a low RIN does not necessarily render RNA unusable. Several studies have highlighted the potential of DV200 as a reliable metric for assessing the RNA quality required for NGS-based genetic studies using FFPE tissue samples [20]. For example, Hatanaka et al. reported that in ODxTT, an amplicon sequencing-based NGS assay for thyroid carcinoma covered by insurance in Japan, DV200 serves as a more effective predictor for detecting gene transcripts than RIN [8]. The study further noted that although the qPCR method is preferred for the quality assessment of ODxTT, DIN, and DV200 serve as valuable alternatives when the qPCR method is not feasible. Electrophoresis-based assessments such as DIN and DV200 offer practical and cost-effective options for nucleic acid quality assessment because of their accessibility and low cost, making them suitable for facilities with limited budgets [8].

The differential effects of formalin fixation on DNA and RNA quality present a challenge in optimizing sample preparation for thyroid cancer genomic analysis, where both DNA-based (e.g., *BRAF* V600E mutations) and RNA-based (e.g., *RET* and *NTRK* fusion genes) analyses are crucial. Our findings suggest that separately fixed tumor samples may offer a solution by enabling thorough fixation in a shorter time frame, potentially preserving both DNA and RNA quality more effectively for comprehensive genomic profiling. Additionally, our study explored the relationship between histological features and nucleic acid quality in thyroid carcinoma specimens, revealing complex interactions that vary with the specimen type. In thyroid gland tumors, cystic changes correlate with an improved S/L Ct ratio, suggesting that formalin injection may facilitate better local penetration in cystic areas. Conversely, an increased lymphocyte ratio was associated with a decreased RIN and DV200, indicating potential RNA quality degradation in areas with high lymphocyte infiltration. This observation raises questions about whether the lower RNA quality is due to the intrinsic properties of infiltrating lymphocytes or their effects on the surrounding tissue. However, to our knowledge, limited literature has directly addressed this relationship in thyroid carcinoma, highlighting an area for future research.

Regarding lymph node metastases, multiple regression analysis revealed that the fibrosis ratio negatively impacted the S/L Ct ratio, suggesting that fibrotic tissue may hinder fixative penetration. Furthermore, macrodissection is associated with improved RIN. However, given the small number of macrodissection cases (*n* = 3), these findings should be interpreted with caution. In contrast, separately fixed tumor samples showed less of an overall influence of histological features. Nevertheless, the presence of cystic components significantly positively affected the DIN and S/L Ct ratio, which is consistent with observations in thyroid gland tumors. No significant differences in nucleic acid quality were observed between BRAF immunohistochemistry-positive and immunohistochemistry-negative samples across all specimen types. These findings demonstrate that the nucleic acid quality in thyroid carcinoma specimens is influenced by histological features and specimen processing methods.

**Fig. 5:**
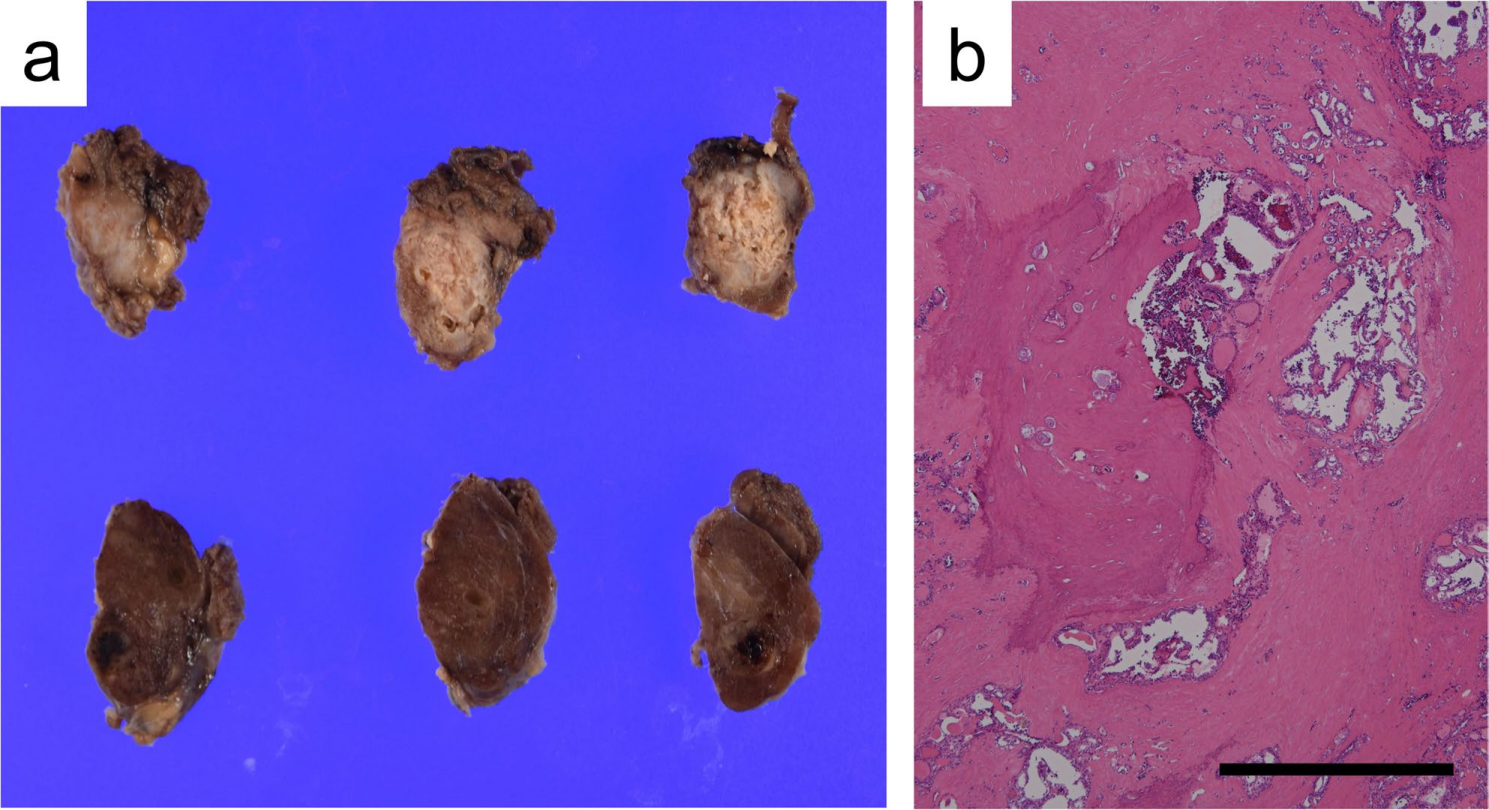
Macroscopic and microscopic images of papillary thyroid carcinoma with *ETV6*-*NTRK3* fusion gene. **a** Macroscopic findings of the cut surface of the thyroid gland in a case of papillary thyroid carcinoma with *ETV6-NTRK3* fusion. Extensive calcification was observed in the tumor area, accompanied by marked sclerosis. **b** Microscopic findings of papillary thyroid carcinoma with *ETV6-NTRK3* fusion gene. Prominent coarse calcifications are observed against a background of extensive fibrosis. Hydrochloric acid-based decalcification was required for slide preparation for diagnosis, although it is known to decrease nucleic acid quality (scale bar = 1000 µm). Note that this figure is included for illustrative purposes only and does not represent the cases analyzed in this study

Finally, the significance of using lymph node metastases specimens for genomic analysis should be highlighted. When lymph node metastases are used as specimens for genomic analysis, it is important to determine whether the genetic mutations in lymph node metastases are identical to or greater than those in the primary tumor. Previous studies have reported that genetic alterations in primary tumors and lymph node metastases exhibit differences and similarities, depending on the type of cancer [23–25]. However, *BRAF* mutations and *RET* and *NTRK* fusions, major markers for molecularly targeted therapies in thyroid carcinoma [26–28], are considered early molecular events in thyroid carcinogenesis [1, 29–31], which may indicate their presence in lymph node metastases similar to their primary lesions. Therefore, if the quality of nucleic acids is equivalent to or greater than that in the primary tumors and the number of tumor cells and the tumor content ratio meet the thresholds for standard genomic analysis, it would be acceptable to consider lymph node metastases as candidates for genomic analysis in thyroid carcinoma. In addition, we discuss the characteristics of lymph node metastasis in thyroid carcinomas. Based on our clinical experience, lymph node metastases in thyroid cancer often exhibit a regional growth pattern and tend not to intermingle extensively with the background lymphocytes. This characteristic potentially facilitates the acquisition of samples with a high tumor content, especially when combined with macrodissection techniques, making them particularly suitable for genomic analysis. Our previous studies indicated that the calcification of lymph node metastases, particularly in cases of PTC with *RET* or *NTRK* fusions, is often mild or absent, even when the thyroid gland itself exhibits significant calcification (Fig. 5) [32–34]. Decalcification methods are known to significantly damage nucleic acids in FFPE tissue samples [32, 34, 35]. Considering that the nucleic acid quality of lymph node metastases is comparable to that of thyroid gland tumors, these metastases can be considered valuable specimens for genomic analysis, especially given their lower degree of calcification. However, our findings also revealed that the quality of nucleic acids in lymph node metastases tended to decrease with increasing metastasis size. Consequently, surgeons, pathologists, and/or laboratory technicians may be required to excise or collect separately fixed tumor samples from large lymph node metastases to preserve the quality of nucleic acids for analysis.

In conclusion, the preparation of separately fixed tumor samples is an effective method for preserving DNA and RNA quality for genetic testing. Specimen collection using biopsy punches is feasible in many facilities, even when managing frozen specimens. These results will contribute to the establishment of a method for preserving high-quality pathology specimens that are widely used in general facilities. However, considering the burden of preparing additional samples, it is important for clinicians, pathologists, and laboratory technicians to appropriately assess the necessity for a sample. This can be facilitated by ensuring that the pathology request form includes detailed information on the potential requirements for future genetic testing, TNM classification, and any other relevant clinical observations. Clinicians and pathologists should share this information in advance. Additionally, our findings revealed that lymph node metastases often exhibit nucleic acid quality equal to or superior to that of thyroid gland tumors, underscoring their potential as reliable sources for genomic analysis.

### Limitations

This study has several limitations. First, the efficacy of negative-pressure fixation in enhancing formalin permeation and preserving nucleic acid quality has not yet been conclusively established. Additionally, the stability of nucleic acid quality in separately fixed tumor samples over time remains to be thoroughly investigated. Electrophoretic assessments, such as RIN and DV200, were used for RNA quality evaluation because of the lack of established qPCR protocols for analyzing degraded RNA in our hospital. Furthermore, this study primarily focused on papillary thyroid carcinoma, which may limit the applicability of our findings to other histological types of thyroid carcinomas, such as follicular, poorly differentiated, and anaplastic carcinomas. Additionally, non-neoplastic benign thyroid lesions were not included in this study. This study used 3–5 mm biopsy punch needles, similar to those used in another ongoing study in lung cancer research. We cannot rule out the possibility that different diameters may be advantageous in some situations. While frozen tissue samples were collected, detailed information about their quality is lacking because processing was conducted in a different division of our institution.

## Supplementary Information

Below is the link to the electronic supplementary material.

Supplementary file1 (DOCX 48 KB)

## Data Availability

The datasets used and/or analyzed in this study are available from the corresponding author upon reasonable request.
