# Supplementary material for: Effective Preparation of FFPE Tissue Samples for Preserving Appropriate Nucleic Acid Quality for Genomic Analysis in Thyroid Carcinoma
Source: Endocr Pathol. 2024 Nov 19;35(4):372–84. doi: 10.1007/s12022-024-09838-9 (PMC11659341; doi:10.1007/s12022-024-09838-9)
Supplement: Supplementary file 1 — Supplementary file1 (DOCX 48 KB) [file 12022_2024_9838_MOESM1_ESM.docx]

Supplementary Table 1. Detailed clinicopathological characteristics of 54 thyroid carcinoma cases

| Case number | Age | Sex | Histological Type | pT | pN | cM | Surgical Procedure | Thyroid gland tumor size (mm) | Max LN metastasis size (mm) | Incision for large LN metastases | BRAF IHC | Thyroid and LN  fixation time (hours) | Separately fixed tumor sample  fixation time (hours) |
| --- | --- | --- | --- | --- | --- | --- | --- | --- | --- | --- | --- | --- | --- |
| 1 | 54 | Female | PTC | pT1b | pN1b | cM1 (lung) | Thyroidectomy with lymphadenectomy | 11 | 51 | Yes | Positive | 41.7 | 17.8 |
| 2 | 62 | Female | PTC (recurrent) | Residual recurrence (pT1 level) | pN1b | cM1 (lung) | Completion of thyroidectomy with lymphadenectomy | 6 | 14 | Yes | Positive | 45.1 | 23.9 |
| 3 | 48 | Male | PTC-Follicular Variant | pT3b | pN1b | cM0 | Thyroidectomy with lymphadenectomy | 35 | 34 | No | Negative | 66.3 | 18.8 |
| 4 | 32 | Female | PTC | pT2 | pN1a | cM0 | Thyroidectomy with lymphadenectomy | 21 | 6 | No | Positive | 63.9 | 16.5 |
| 5 | 71 | Female | FTC | pT3b | pN1b | cM0 | Thyroidectomy with lymphadenectomy | 61 | 25 | No | Negative | 42.1 | 17.1 |
| 6 | 43 | Female | PTC-Solid variant | pT3a | pN1a | cM0 | Thyroidectomy with lymphadenectomy | 41 | 4 | No | Negative | 68.8 | 22.3 |
| 7 | 76 | Male | PTC | pT2 | pN1b | cM0 | Thyroidectomy with lymphadenectomy | 24 | 12 | No | Positive | 38.5 | 18.3 |
| 8 | 29 | Female | PTC | pT1b | pN1a | cM0 | Thyroidectomy with lymphadenectomy | 14 | 3 | No | Positive | 69.3 | 22.5 |
| 9 | 68 | Female | PTC | pT4a | pN1a | cM0 | Thyroidectomy with lymphadenectomy | 24 | 0.4 | No | Positive | 45.2 | 20.4 |
| 10 | 60 | Female | PTC | pT3b | pN1a | cM0 | Thyroidectomy with lymphadenectomy | 24 | 1.7 | No | Positive | 29.6 | 22.0 |
| 11 | 70 | Female | PTC | pT3b | pN0 | cM0 | Thyroidectomy with lymphadenectomy | 41 |  | No | Positive | 92.9 | 21.1 |
| 12 | 46 | Female | PTC | pT3a | pN0 | cM0 | Thyroidectomy with lymphadenectomy | 54 |  | No | Negative | 41.5 | 17.7 |
| 13 | 52 | Female | PTC | pT1b | pN1a | cM0 | Thyroidectomy with lymphadenectomy | 12 | 0.07 | No | Positive | 77.6 | 21.9 |
| 14 | 47 | Female | PTC | pT3b | pN1a | cM0 | Thyroidectomy with lymphadenectomy | 12 | 11 | No | Positive | 30.0 | 20.6 |
| 15 | 37 | Female | PTC | N/A | pN1b | cM1 (lung) | Lymph node excision only |  | 6 | Yes | Positive | 45.7 | 22.0 |
| 16 | 29 | Male | PTC | Residual recurrence (pT2 level) | pN1b | cM1 (lung) | Completion of thyroidectomy with lymphadenectomy | 27 | 45 | Yes | Negative | 24.9 | 17.1 |
| 17 | 65 | Male | PTC | pT3a | pN1b | cM1 (lung, bone) | Thyroidectomy with lymphadenectomy | 43 | 51 | Yes | Positive | 29.1 | 21.4 |
| 18 | 64 | Female | PDTC | N/A | N/A | cM1 (Cervical LN) | Lymph node excision only |  | 12 | Yes | Positive | 68.4 | 20.6 |
| 19 | 65 | Male | PTC | pT1b | pN0 | cM0 | Thyroidectomy with lymphadenectomy | 16 |  | No | Positive | 22.5 | 22.6 |
| 20 | 42 | Male | PTC | pT1b | pN0 | cM0 | Thyroidectomy with lymphadenectomy | 12 |  | No | Positive | 25.4 | 17.6 |
| 21 | 67 | Male | PTC | pT3b | pN1b | cM1 (lung) | Thyroidectomy with lymphadenectomy | 51 | 21 | Yes | Positive | 92.9 | 21.1 |
| 22 | 64 | Female | PTC | N/A | pN1b | cM0 | Lymph node excision only |  | 24 | Yes | Positive | 29.7 | 21.8 |
| 23 | 75 | Male | PTC-Follicular Variant | N/A | pN1b | cM0 | Lymph node excision only |  | 13 | Yes | Negative | 65.7 | 18.0 |
| 24 | 63 | Female | PTC | pT1b | pN1a | cM0 | Thyroidectomy with lymphadenectomy | 15 | 1.2 | No | Positive | 30.0 | 21.7 |
| 25 | 25 | Male | PTC | pT1b | pN1a | cM0 | Thyroidectomy with lymphadenectomy | 12 | 9 | Yes | Positive | 44.1 | 19.9 |
| 26 | 38 | Female | PTC | pT1b | pN1a | cM0 | Thyroidectomy with lymphadenectomy | 15 | 3 | No | Negative | 25.6 | 22.7 |
| 27 | 75 | Female | PTC | pT1b | pN0 | cM0 | Thyroidectomy with lymphadenectomy | 15 |  | No | Positive | 69.5 | 22.1 |
| 28 | 58 | Male | PTC | pT3b | pN1b | cM0 | Thyroidectomy with lymphadenectomy | 27 | 75 | Yes | Positive | 43.6 | 22.0 |
| 29 | 77 | Female | PTC | Residual recurrence (pT1 level) | pN0 | cM1 (Cervical LN) | Completion of thyroidectomy with lymphadenectomy | 10 |  | No | Positive | 91.7 | 20.8 |
| 30 | 45 | Female | PTC | pT2 | pN1a | cM0 | Thyroidectomy with lymphadenectomy | 28 | 4 | No | Positive | 41.5 | 19.0 |
| 31 | 79 | Female | PTC | Residual recurrence (pT1 level) | pN0 | cM1 (Cervical LN) | Completion of thyroidectomy with lymphadenectomy | 8 |  | No | Positive | 30.0 | 23.0 |
| 32 | 49 | Female | PTC | pT3a | pN1b | cM0 | Thyroidectomy with lymphadenectomy | 62 | 17 | No | Positive | 22.1 | 22.3 |
| 33 | 64 | Male | PTC | N/A | pN1b | cM0 | Lymph node excision only |  | 18 | Yes | Positive | 24.8 | 21.1 |
| 34 | 64 | Male | PTC | pT2 | pN1a | cM0 | Thyroidectomy with lymphadenectomy | 25 | 1.2 | No | Positive | 29.3 | 22.3 |
| 35 | 47 | Female | PTC | pT1b | pN1a | cM0 | Thyroidectomy with lymphadenectomy | 14 | 4 | No | Negative | 93.4 | 22.3 |
| 36 | 54 | Male | PTC | Residual recurrence (pT1 level) | pN1a | cM0 | Completion of thyroidectomy with lymphadenectomy | 9 | 2.3 | No | Positive | 68.7 | 21.9 |
| 37 | 76 | Female | PTC | N/A | pN1b | cM1 (Extracervical LN) | Lymph node excision only |  | 27 | Yes | Positive | 25.5 | 18.1 |
| 38 | 74 | Male | PTC | pT3a | pN1a | cM0 | Thyroidectomy with lymphadenectomy | 42 | 0.28 | No | Positive | 29.4 | 22.0 |
| 39 | 32 | Male | PTC | pT3a | pN1a | cM0 | Thyroidectomy with lymphadenectomy | 42 | 8 | No | Negative | 29.7 | 21.9 |
| 40 | 24 | Male | PTC | Residual recurrence (pT1 level) | pN1b | cM0 | Completion of thyroidectomy with lymphadenectomy | 12 | 10 | Yes | Negative | 68.1 | 20.0 |
| 41 | 66 | Male | PTC | pT1b | pN1a | cM0 | Thyroidectomy with lymphadenectomy | 11 | 2.2 | No | Positive | 29.3 | 22.1 |
| 42 | 75 | Female | PTC | pT4a | pN1a | cM0 | Thyroidectomy with lymphadenectomy | 24 | 5 | Yes | Positive | 27.0 | 22.1 |
| 43 | 35 | Female | PTC | pT2 | pN1a | cM0 | Thyroidectomy with lymphadenectomy | 21 | 2.5 | Yes | Positive | 68.4 | 21.3 |
| 44 | 69 | Male | PTC | Residual recurrence (pT1 level) | pN1b | cM1 (skin) | Completion of thyroidectomy with lymphadenectomy | 15 | 40 | Yes | Positive | 20.4 | 20.3 |
| 45 | 43 | Female | PTC | pT1b | pN1a | cM0 | Thyroidectomy with lymphadenectomy | 15 | 6 | No | Positive | 69.7 | 22.3 |
| 46 | 35 | Female | PTC | pT1b | pN1a | cM0 | Thyroidectomy with lymphadenectomy | 11 | 12 | No | Positive | 75.7 | 21.3 |
| 47 | 32 | Female | PTC | pT1b | pN1a | cM0 | Thyroidectomy with lymphadenectomy | 5 | 4 | No | Positive | 39.1 | 15.7 |
| 48 | 56 | Male | PTC | pT2 | pN1b | cM0 | Thyroidectomy with lymphadenectomy | 21 | 25 | Yes | Negative | 44.6 | 20.9 |
| 49 | 60 | Female | PTC | N/A | N/A | cM1 (Cervical LN, lung) | Lymph node excision only |  | 15 | Yes | Positive | 20.3 | 21.2 |
| 50 | 81 | Female | ATC | pT4a | pN1a | cM0 | Thyroidectomy with lymphadenectomy | 45 | 9 | No | Positive | 27.8 | 20.9 |
| 51 | 76 | Male | PTC | N/A | pN1b | cM0 | Lymph node excision only |  | 42 | Yes | Positive | 28.1 | 21.4 |
| 52 | 54 | Female | PTC | Residual recurrence (pT1 level) | pN0 | cM0 | Completion of thyroidectomy with lymphadenectomy | 11 |  | No | Negative | 66.2 | 18.8 |
| 53 | 68 | Female | PTC | Absence of residual tumor and recurrence (pT0) | pN1b | cM0 | Completion of thyroidectomy with lymphadenectomy |  | 31 | Yes | Positive | 39.4 | 21.4 |
| 54 | 73 | Female | FTC | pT2 | pN0 | cM1 (bone) | Thyroidectomy with lymphadenectomy | 31 |  | No | Negative | 75.5 | 21.7 |

PTC: Papillary thyroid carcinoma; FTC: Follicular thyroid carcinoma; PDTC: Poorly differentiated thyroid carcinoma; ATC: Anaplastic thyroid carcinoma; LN: Lymph node; IHC: Immunohistochemistry; N/A: Not applicable.

Clinicopathological characteristics of 54 thyroid carcinoma cases. Tumor sizes and lymph node metastasis sizes are maximum diameters. Incisions for large LN metastases indicate whether incisions were made to enhance formalin penetration. BRAF IHC shows the result of BRAF V600E immunohistochemistry. Fixation times represent the duration of formalin immersion for main specimens (thyroid glands and lymph nodes) and separately fixed tumor samples, respectively.

Supplementary Table 2. Comprehensive analysis of associations between histological features and nucleic acid quality parameters

| Specimen type | Feature | DIN | S/L Ct ratio | RIN | DV200 |
| --- | --- | --- | --- | --- | --- |
| Thyroid gland tumors | Tumor content | P=0.200 / P=0.070 | P=0.665 / P=0.464 | P=0.117 / P=0.333 | P=0.606 / P=0.728 |
|  | Fibrosis ratio | P=0.840 / P=0.352 | P=0.661 / P=0.467 | P=0.665 / P=0.366 | P=0.227 / P=0.976 |
|  | Lymphocyte ratio | P=0.527 / P=0.450 | P=0.270 / P=0.636 | P=0.070 / P=0.038 (r=-0.310) | P=0.013 (β=-0.535) / P=0.032 (r=-0.320) |
|  | Cystic change | P=0.266 / P=0.128 | P=0.038 (β=0.373) / P=0.049 | P=0.564 / P=0.367 | P=0.524 / P=0.47 |
|  | Macrodissection | P=0.648 / P=0.365 | P=0.658 / P=0.398 | P=0.573 / P=0.313 | P=0.428 / P=0.677 |
| Lymph node metastases | Tumor content | P=0.832 / P=0.002 (r=-0.469) | P=0.059 / P=0.018 (r=-0.364) | P=0.484 / P=0.463 | P=0.250 / P=0.003 (r=-0.451) |
|  | Fibrosis ratio | P=0.519 / P=0.019 (r=-0.362) | P=0.001 (β=-0.705) / P=0.102 | P=0.880 / P=0.958 | P=0.114 / P=0.715 |
|  | Lymphocyte ratio | P=0.376 / P<0.001 (r=0.534) | P=0.095 / P=0.011 (r=0.388) | P=0.405 / P=0.467 | P=0.054 / P=0.010 (r=0.395) |
|  | Cystic change | P=0.333 / P=0.432 | P=0.552 / P=0.333 | P=0.825 / P=0.651 | P=0.583 / P=0.810 |
|  | Macrodissection | P=0.706 / P=0.198 | P=0.293 / P=0.275 | P=0.010 (β=0.427) / P=0.041 | P=0.110 / P=0.026 |
| Separately fixed tumor samples | Tumor content | P=0.08 / P=0.25 | P=0.569 / P=0.59 | P=0.943 / P=0.795 | P=0.555 / P=0.529 |
|  | Fibrosis ratio | P=0.51 / P=0.354 | P=0.875 / P=0.566 | P=0.670 / P=0.858 | P=0.379 / P=0.404 |
|  | Lymphocyte ratio | P=0.657 / P=0.485 | P=0.676 / P=0.297 | P=0.629 / P=0.457 | P=0.317 / P=0.879 |
|  | Cystic change | P=0.001 (β=0.419) / P=0.004 | P=0.011 (β=0.346) / P=0.015 | P=0.788 / P=0.606 | P=0.769 / P=0.987 |
|  | Macrodissection | P=0.76 / P=0.5 | P=0.667 / P=0.749 | P=0.708 / P=0.415 | P=0.371 / P=0.55 |

DIN: DNA integrity number; S/L Ct ratio: short-to-long cycle threshold ratio; RIN: RNA integrity number.

Values are presented as *P*-values for multiple regression analysis and univariate analysis (Spearman correlation for continuous variables, Mann–Whitney U test for categorical variables). Significant associations (*P* < 0.05) are shown in bold. For significant associations, standardized β coefficients (multiple regression) or correlation coefficients (r) (univariate analysis) are shown in parentheses.
